# Follistatin-like protein 1 functions as a potential target of gene therapy in proliferative diabetic retinopathy

**DOI:** 10.18632/aging.202678

**Published:** 2021-03-10

**Authors:** Rui Niu, Ze-Tong Nie, Lin Liu, Yu-Wen Chang, Jian-Qun Shen, Qiong Chen, Li-Jie Dong, Bo-Jie Hu

**Affiliations:** 1 Tianjin Key Laboratory of Retinal Functions and Diseases, Tianjin International Joint Research and Development Centre of Ophthalmology and Vision Science, Eye Institute and School of Optometry, Tianjin Medical University Eye Hospital, Tianjin, China; 2 Hetian District People's Hospital, Xinjiang, China

**Keywords:** proliferative diabetic retinopathy, VEGF, FSTL1, CTGF, fibrosis

## Abstract

The degree of retinal fibrosis increased in proliferative diabetic retinopathy (PDR) patients after administration of anti-Vascular endothelial growth factor (VEGF) injections. Previous studies showed that the balance between connective tissue growth factor (CTGF) and VEGF plays an important role. Therefore, in a high-glucose state, an anti-VEGF and CTGFshRNA dual-target model was used to simulate clinical dual-target treatment in PDR patients, and RNA sequencing (RNA-Seq) technology was used for whole transcriptome sequencing. A hypoxia model was constructed to verify the sequencing results at the cellular level, and the vitreous humor and proliferative membranes were collected from patients for verification. All sequencing results included Follistatin-like protein 1 (*FSTL1*) and extracellular matrix (ECM) receptor pathway, indicated that anti-VEGF therapy may upregulate FSTL1 expression, while dual-target treatment downregulated FSTL1. Thus, we further studied the function of FSTL1 on the expression of VEGF and ECM factors by both overexpressing and silencing *FSTL1*. In conclusion, our results suggested that FSTL1 may be involved in the pathogenesis of PDR and is related to fibrosis caused by the anti-VEGF treatment, thus providing a potential target for gene therapy in PDR.

## INTRODUCTION

Diabetic retinopathy is the most important manifestation of diabetic microangiopathy and a major cause of adult visual impairment [1]. Proliferative diabetic retinopathy (PDR) is a progressive disease caused by retinal neovascularization, and its hallmark features include retinal neovascularization and fibrovascular membrane [2]. Vascular endothelial growth factor (VEGF) is a unique and potent angiogenic factor that stimulates angiogenesis [3]. In recent years, intravitreal injection of anti-VEGF has been used as an adjunct to vitrectomy in patients with PDR, by limiting bleeding from new vessels intraoperatively and reducing bleeding when peeling the membranes [4].

Many studies have shown that anti-VEGF treatment can inhibit neovascularization but also increase connective tissue growth factor (CTGF) expression, aggravate fibrotic processes, and promote real-time dynamic conversion of CTGF and VEGF [5–7]. The ratio of CTGF / VEGF is a strong predictor of vascular and fibrosis transformation [8]. We later showed microvascular ultrastructural changes were detected in PDR patients treated with anti-VEGF and anti-CTGF dual-target therapy, and these findings were supported in a diabetic rodent model [9].

Therefore, we analyzed differentially expressed genes between dual-target treatment (anti-VEGF + CTGFshRNA) and anti-VEGF treatment based on RNA-Seq technology to search for potential molecular mechanisms in dual-target therapy. Among the comparison groups, the common differential genes included *MET*, *COL11A1*, *ITGBL1*, *FN1*, *LGR5*, *UGCG*, *SPP1*, *EIF2AK3*, and *Fstl1*. Based on previous literatures, we determined that FSTL1 not only plays a role in fibrosis but also involves in angiogenesis [10, 11]. We believe that FSTL1 may be a new therapeutic target for the treatment of both neovascularization and fibrosis in PDR. Thereafter, we verified the results by analyzing samples obtained from clinical patients using enzyme-linked immunosorbent assays (ELISA) and immunofluorescence. We further evaluated the expression of FSTL1 in HRCECs and HUVECS under hypoxic conditions using immunofluorescence, qPCR and western blots[12]. We constructed *FSTL1* expression plasmids and an *FSTL1* interference fragment, respectively, to transfect HUVECs to clarify the effect of FSTL1 on vascular endothelial cells by overexpressing or silencing *FSTL1* expression. Further study of potential molecular mechanisms is required to provide new targets for the development of novel intervention tools. We hope to find a new treatment method to solve the problems of neovascularization and fibrosis simultaneously, so as to provide theoretical and experimental basis for the treatment of PDR.

## RESULTS

### Intravitreal bevacizumab (IVB) injection accelerated fibrotic proliferative retinopathy

**Figure 1:**
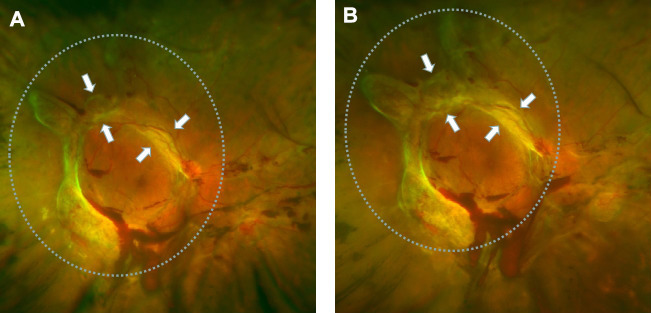
**Retinal fibrosis accelerated after IVB treatment.** (**A**) The fundus photograph shows pre-retinal hemorrhage and fibrosis in the macula and optic disc regions of the retina, a circular proliferative membrane presence before IVB treatment. (**B**) Retinal fibrosis was obviously accelerated post-IVB treatment. The arrows indicate the progression of pre-retinal membrane fibrosis compared to pretreatment conditions.

Many studies have proved that intravitreal anti-VEGF injection could increase the severity of fibrosis in patients. We have frequently observed this phenomenon in clinical settings. At present, there are no effective treatments for this phenomenon, which rationalizes further research on the subject. As shown in Figure 1, fundus photographs of a patient with PDR were acquired before and after intravitreal bevacizumab injection (IVB). The image before IVB treatment showed a proliferative retinal anterior membrane in the upper retina, while IVB treatment accelerated fibrosis.

### Immunofluorescence analysis of proliferation membrane from PDR patients with and without anti-VEGF

**Figure 2:**
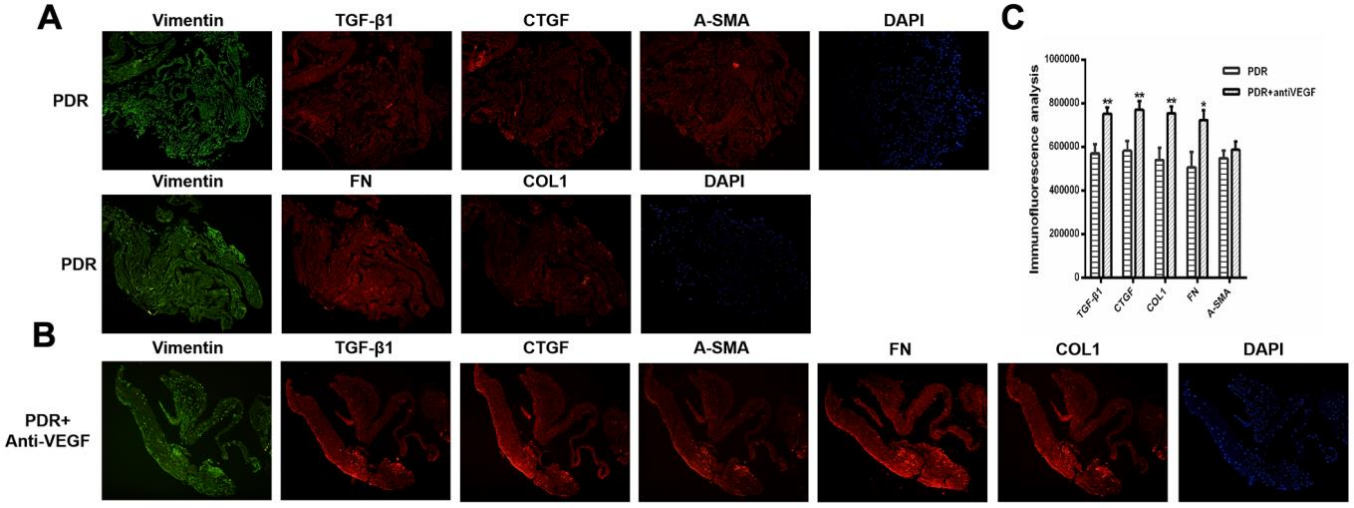
**TGFβ1, CTGF, COL1, and FN were upregulated in the membrane after antiVEGF-treated.** (**A**) TGFβ1, CTGF, α-SMA, FN and COL1 proliferative membrane immunofluorescence results in PDR patients without anti-VEGF treatment. (**B**) TGFβ1, CTGF, α-SMA, FN and COL1 proliferative membrane immunofluorescence results in PDR patients with anti-VEGF treatment. Vimentin, as an internal reference. DAPI, the tracer shows the nuclear position of the fluorescent agent 4',6-diamidino-2-phenylindole (magnification: ×10). (**C**) The levels of TGFβ1, CTGF, COL1, FN, and α-SMA were analyzed using ImageJ. Expression of TGFβ1, CTGF, COL1, and FN were significantly higher in the membranes obtained from antiVEGF-treated PDR patients than in the membranes obtained from untreated PDR patients. Data are expressed as mean ± SEM. * means P < 0.05, **means P < 0.01, *** means P < 0.001.

Based on the above finding, proliferative epiretinal membranes from PDR patients with or without anti-VEGF treatment were collected to identify the fibrosis activities of anti-VEGF at the molecular level. We detected pro-fibrotic factor CTGF and other fibrosis-related factors, such as vimentin, transforming growth factor - beta 1 (TGFβ1), fibronectin (FN), α-smooth muscle actin (α-SMA), and collagen 1 (COL1) during immunofluorescence analysis. Specific binding was observed at the proliferative membrane in PDR patients with and without anti-VEGF treatment, with the specificity indicating the degree of factor binding (magnification: ×10). We observed positive labelling for vimentin, TGFβ1, CTGF, FN, α-SMA, and COL1 in all fibrotic membrane samples (Figure 2). Levels of TGFβ1, CTGF, FN, α-SMA, and COL1 were analyzed using ImageJ. IVB-treated membranes strongly expressed TGFβ1, CTGF, FN and COL1 compared with membranes obtained from PDR patients without anti-VEGF treatment, exhibiting statistically significant differences (*p <*0.05, Figure 2C).

### Differential gene analysis

In order to further study the molecular mechanism through which fibrosis was induced by anti-VEGF under high glucose. We used RNA-Seq technology to determine the genes that were differentially expressed between the dual-target treatment (anti-VEGF + CTGFshRNA) and the anti-VEGF treatment to search for the potential molecular mechanisms involved in dual-target therapy [12].

**Figure 3:**
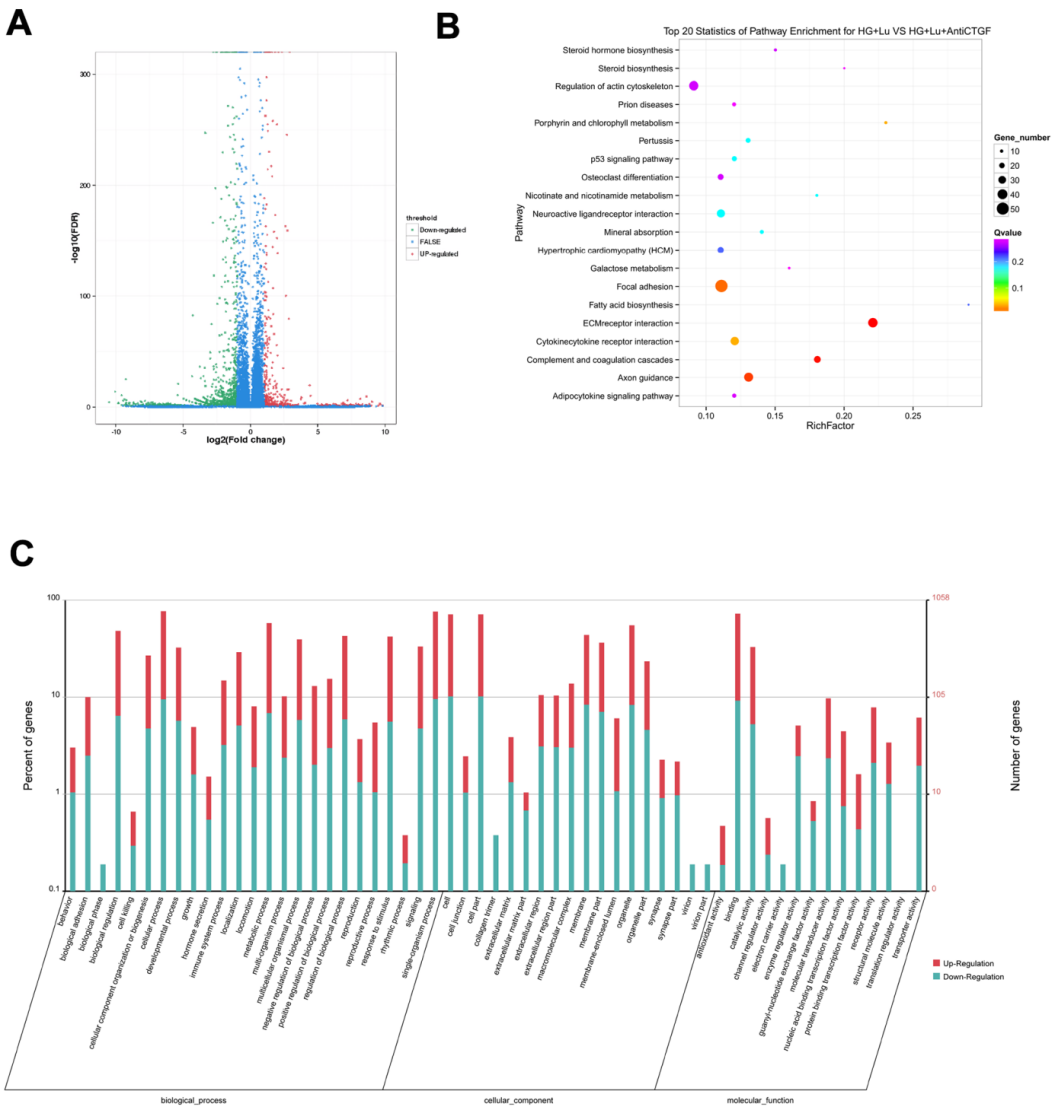
**Differential gene analysis in the HG + Lu and HG + Lu + anti-CTGF groups.** (**A**) A volcano map showed the differentially expressed genes in cell samples of the HG + Lu and HG + Lu + anti-CTGF groups. The blue dots indicate the genes whose expression was not significantly different between the two groups; the red dots indicate the significantly upregulated genes in the HG + Lu + anti-CTGF group as compared to HG + Lu group; the green dots refer to the significantly downregulated genes. (**B**) KEGG pathway analysis indicated the top 20 signaling pathways enriched by the differentially expressed genes in the HG + Lu and HG + Lu + anti-CTGF enrichment comparison. The x-axis represents the ratio of candidate genes to the background gene. The y-axis represents the GO terms. The color of the bubble corresponds to the enrichment (-log10 (*P* value)), and the size of the bubble is proportional to the number of genes enriched in the pathway. (**C**) HG + Lu and HG + Lu + anti-CTGF differential gene GO function annotation classification. The GO enrichment analysis revealed that the differentially expressed genes were enriched in biological processes, cellular components, and molecular functions. The x-axis represents differentially expressed genes in biological processes, cellular components, and molecular functions. The y-axis on the left represents the percentage of genes that are up- or downregulated, and the y-axis on the right represents the number of genes. Red represents upregulated genes, and blue represents downregulated genes.

**Figure 4:**
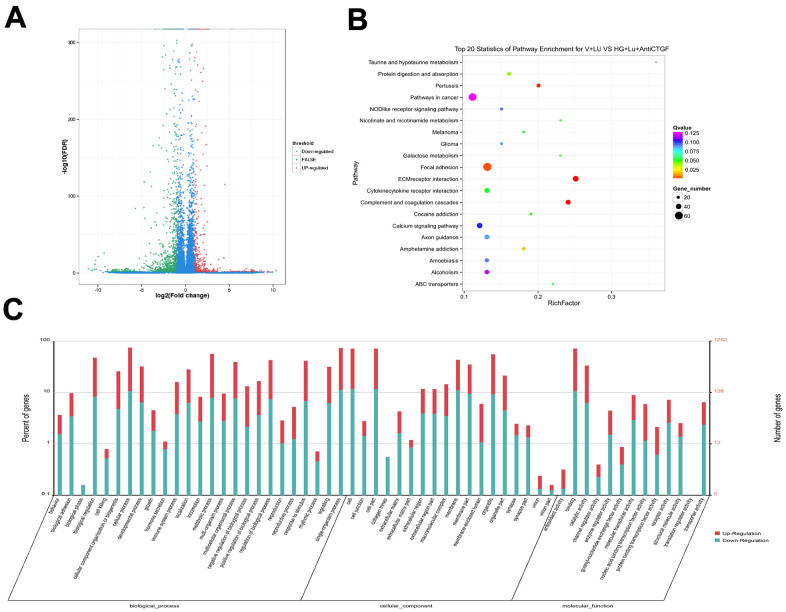
**Differential gene analysis in the VEGF + Lu and HG + Lu + anti-CTGF groups.** (**A**) A volcano map showed the differentially expressed genes in cell samples of the VEGF + Lu and HG + Lu + anti-CTGF groups. The blue dots indicate the genes whose expression was not significantly different between the two groups; the red dots indicate the significantly upregulated genes in the HG + Lu + anti-CTGF group as compared to the VEGF + Lu group; the green dots refer to the significantly downregulated genes. (**B**) KEGG pathway analysis indicated the top 20 signaling pathways enriched by the differentially expressed genes in the VEGF + Lu and HG + Lu + anti-CTGF enrichment comparison. The x-axis represents the ratio of candidate genes to the background gene. The y-axis represents the GO terms. The color of the bubble corresponds to the enrichment (-log10 (*P* value)), and the size of the bubble is proportional to the number of genes enriched in the pathway. (**C**) VEGF + Lu and HG + Lu + anti-CTGF differential gene GO function annotation classification. The GO enrichment analysis revealed that the differentially expressed genes were enriched in biological processes, cellular components, and molecular functions. The x-axis represents differentially expressed genes in biological processes, cellular components, and molecular functions. The y-axis on the left represents the percentage of genes that are up- or downregulated, and the y-axis on the right represents the number of genes. Red represents upregulated genes, and blue represents downregulated genes.

The raw data information, gene coverage and sequencing quality assessment data were shown in the supplementary materials (Supplementary Figure 1 and Supplementary Table 1). Compared with the HG + Lu + anti-CTGF group, 1337 genes were differentially expressed in the HG + Lu group, of which, 918 were upregulated and 419 were downregulated (Table 1, Figure 3A). Compared with the HG + Lu + anti-CTGF group, 1613 genes were differentially expressed in the V + Lu group, of which, 1159 were upregulated and 454 were downregulated (Table 2, Figure 4A). Among the differentially expressed genes (DEGs), those with significantly differential gene expression were selected, and their respective characteristics and expression levels were compared between the two groups. (HG: high glucose, Lu: Lucentis, an anti-VEGF drug, V: VEGF).

**Table 1:** HG + LU and HG + LU + anti-CTGF sequencing quality test results.

**Gene**	**Gene length**	**Log2 ratio (HG + LU + anti-CTGF group / HG + LU group)**	**Up- or downregulated**	**P value**
HMOX1	1465	2.92	Up	<0.01
SPP1	1546	2.12	Up	<0.01
GCLC	5787	2.09	Up	<0.01
LCN2	877	1.48	Up	<0.01
ANTXR2	1455	1.47	Up	<0.01
SQSTM1	3982	1.42	Up	<0.01
FTH1	899	1.41	Up	<0.01
HSPA5	4132	1.32	Up	<0.01
MET	6694	-1.05	Down	<0.01
TXNRD1	3920	1.21	Up	<0.01
ITGBL1	1338	-1.31	Down	<0.01
COL11A1	5457	-1.82	Down	<0.01
FN1	7151	-1.19	Down	<0.01
LGR5	4216	-3.36	Down	<0.01
UGCG	1346	-1.54	Down	<0.01
EIF2AK3	6323	-1.33	Down	<0.01
Fstl1	5903	-1.30	Down	<0.01

**Table 2:** V + LU and HG + LU + anti-CTGF sequencing quality test results.

**Gene**	**Gene length**	**Log2 ratio (HG + LU + anti-CTGF group / V + LU group)**	**Up- or downregulated**	**P value**
MET	6694	-1.03	Down	<0.01
COL11A1	5457	-2.45	Down	<0.01
ITGBL1	1338	-1.31	Down	<0.01
COL12A1	11636	-1.15	Down	<0.01
FN1	7151	-1.98	Down	<0.01
LGR5	4216	-2.48	Down	<0.01
UGCG	1346	-1.90	Down	<0.01
SPP1	1546	2.43	Up	<0.01
EIF2AK3	6323	-1.22	Down	<0.01
Fstl1	5903	-1.51	Down	<0.01

The results showed that these DEGs participate in molecular biological processes and affect the energy metabolism of cells and protein synthesis. We focused our attention on fibrosis-related genes, in which we considered a height difference adjustment of the top 200 DEGs according to *p* value.

The significant functional enrichment analysis of GO functions showed that in the HG + Lu vs. HG + Lu + anti-CTGF group comparison, DEG functions could mainly be divided according to the following three aspects: cell components, molecular functions, and biological processes (Figure 3C). In the V + Lu vs. HG + Lu + anti-CTGF group comparison, DEG functions could be divided into the following three aspects: cell components, molecular functions, and biological processes (Figure 4C). Among the two comparison groups, the common differential genes included *MET*, *COL11A1*, *ITGBL1*, *FN1*, *LGR5*, *UGCG*, *SPP1*, *EIF2AK3*, and *Fstl1*.

*In vivo*, different genes coordinate with each other to perform their biological functions, and pathway-based analysis helps us to further our understanding of the biological functions of genes. In the comparison between the HG + Lu and HG + Lu + anti-CTGF groups (Figure 3B), the significant pathways were mainly involved in ECM-receptor interactions, complement and coagulation cascades, pertussis, focal adhesion, amphetamine addiction, cocaine addiction, cytokine-cytokine receptor interactions, and ABC transporters. In the comparison between the V + Lu and HG + Lu + anti-CTGF groups, the significant pathways were ECM-receptor interactions, complement and coagulation cascades, axon guidance, focal adhesion, cytokine-cytokine receptor interactions, and neuroactive ligand-receptor interactions (Figure 4B). Among them, gene expression in the ECM-receptor interactions pathway attracts the most attention, where the DEGs, such as *FN1*, *SPP1*, *MET*, and *Fstl1*, among others, were closely related to the fibrosis process.

### ELISA detection of FSTL1

Based on previous research and RNA-seq results, we decided to study the role of FSTL1 in DR by detecting the expression of FSTL1 in PDR patients with or without anti-VEGF. We determined the expression of FSTL1 in vitreous humor with and without anti-VEGF using an FSTL1 ELISA kit. The clinical characteristics of the PDR group (15 eyes, 13 patients) and PDR + antiVEGF group (18 eyes, 16 patients) are presented in Table 3. There were no significant differences in the clinical characteristics, including gender, age, years with diabetes, HbA1c, fasting blood glucose, systolic BP and diastolic BP, between the two groups (Table 3).

**Table 3:** Clinical characteristics of the subjects.

	**PDR (n = 13)**	**PDR + antiVEGF (n = 16)**	**Test value**	**P value**
Male/female	4/9	8/8	x^2^ = 1.09	0.31
Age	58.00 ± 2.33	51.63 ± 2.13	t = 2.01	0.05
Years with diabetes (year)	15.46 ± 7.31	12.33 ± 8.30	t = 1.29	0.21
HbA1c (%)	7.31 ± 1.15	7.74 ± 1.65	t = 0.65	0.52
Fasting blood glucose (mmol/L)	7.54 ± 2.74	7.75 ± 2.01	t = 0.12	0.90
Systolic BP (mmHg)	157.31 ± 18.00	143.73 ± 23.65	t = 1.62	0.12
Diastolic BP (mmHg)	78.92 ± 9.50	81.27 ± 12.25	t = 0.17	0.87

**Figure 5:**
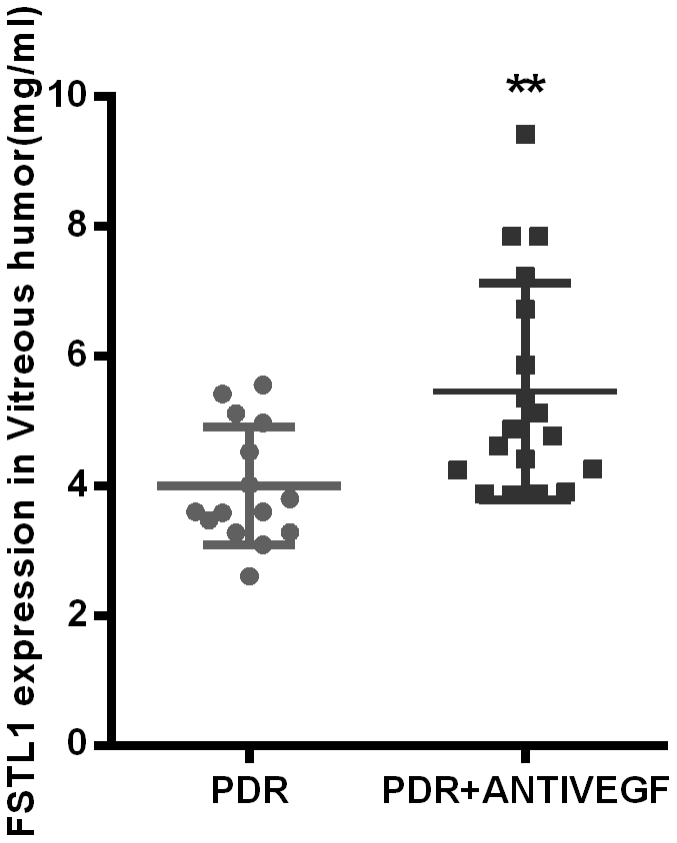
**FSTL1 expressed higher in vitreous humor in the anti-VEGF-treated PDR patients.** The level of FSTL1 expression in vitreous humor was significantly higher in the anti-VEGF-treated PDR patients than in the untreated PDR patients. Data are expressed as mean ± SEM. * means P < 0.05, **means P < 0.01, *** means P < 0.001.

The FSTL1 expression levels in the PDR group and PDR + Anti-VEGF group were 4.37 ± 1.24 ng/mL and 6.06 ± 2.18 ng/mL, respectively, with a statistically significant difference (*p*<0.05, Figure 5).

### Immunofluorescence of fibrotic membrane samples

**Figure 6:**
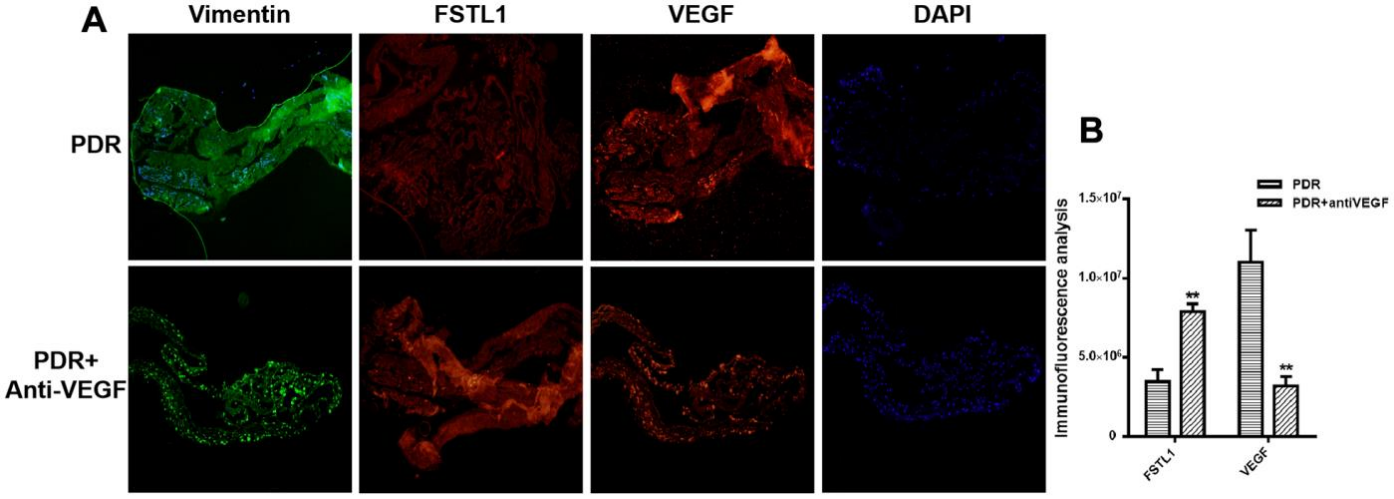
**Stronger FSTL1 staining and weaker VEGF staining in the IVB-treated membranes.** (**A**) FSTL1 and VEGF proliferative membrane immunofluorescence results in PDR patients with and without anti-VEGF treatment. Vimentin, as an internal reference. DAPI, the tracer shows the nuclear position of the fluorescent agent 4',6-diamidino-2-phenylindole (magnification: ×10). (**B**) The levels of FSTL1 and VEGF were analyzed using ImageJ. Stronger FSTL1 staining was detected in the IVB-treated membranes than in the untreated membranes, and VEGF staining was weaker in the IVB-treated group than in the untreated group. Data are expressed as mean ± SEM. * means P < 0.05, **means P < 0.01, *** means P < 0.001.

All fibrotic membrane samples were positive for vimentin, FSTL1, and VEGF. Stronger FSTL1 staining was detected in IVB-treated membranes compared to untreated membranes, while VEGF staining was weaker in the IVB-treated group than in the untreated group (*p<0.05*, Figure 6).

### Expression of FSTL1 under hypoxia

Hypoxia plays an important role in the occurrence and development of retinal vascular disease. The main pathogenesis of PDR is retinal neovascularization induced by high glucose and its associated oxidative stress. Therefore, we evaluated the effects of hypoxia on HRCECs and HUVECs. We divided cells into normal, 3 h hypoxia, and 6 h hypoxia groups. The expression of FSTL1 and ECM-related factors were detected using immunofluorescence, qPCR and western blots.

### The expression of FSTL1 and ECM-related factors in HRCECs under hypoxic conditions

**Figure 7:**
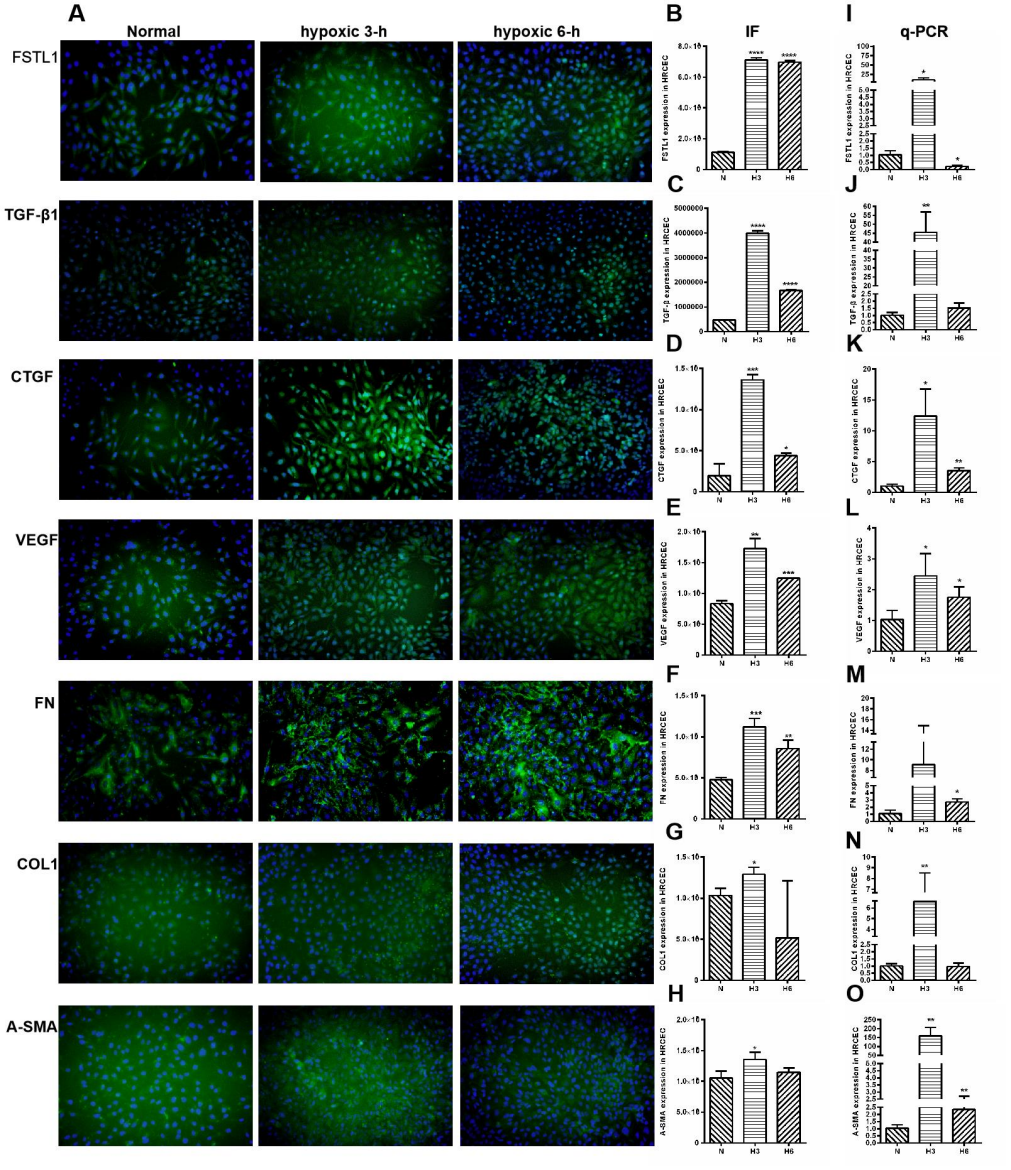
**The expression of FSTL1, TGFβ, CTGF, VEGF, FN, COL1 and α-SMA in HRCECs.** In Immunofluorescence figures, (**A**) indicates normal cell fluorescence, hypoxic 3 h cell fluorescence and hypoxic 6 h cell fluorescence (10×). (**B**–**H**) indicates the quantitative analysis of immunofluorescence figures in the normal, 3 h hypoxia, and 6 h hypoxia models using ImageJ. The 3 h and 6 h hypoxia results indicated that FSTL1, TGFβ1, CTGF, VEGF, FN, COL1, and α-SMA were expressed at significantly higher levels than in normal cells. (**I**–**O**) indicates that the mRNA expression of *FSTL1* and ECM-related factors in the three models. The expression of *FSTL1*, *TGFβ1*, *CTGF*, *VEGF*, *FN*, *COL1*, and *α-SMA* mRNA in 3 h and 6 h hypoxia conditions, respectively, were expressed at significantly higher levels than in normal cells (N: normal, h3: 3 h hypoxia, h6: 6 h hypoxia). Data are expressed as mean ± SEM. * means P < 0.05, **means P < 0.01, *** means P < 0.001.

The expression of FSTL1 and ECM-related factors were analyzed under hypoxic conditions and the results provided a foundation for subsequent FSTL1-related experiments. In the hypoxic model, which included hypoxia for 3 hours and 6 hours, FSTL1, TGFβ1, CTGF, VEGF, FN, COL1, and α-SMA were expressed at significantly higher levels than were observed in normal HRCECs (immunofluorescence, Figure 7A), (*p*<0.05, Figure 7B–7H). These results were consistent with qPCR results (*p*<0.05, Figure 7I–7O).

### The expression of FSTL1 and ECM-related factors in HUVECs under hypoxic conditions

**Figure 8:**
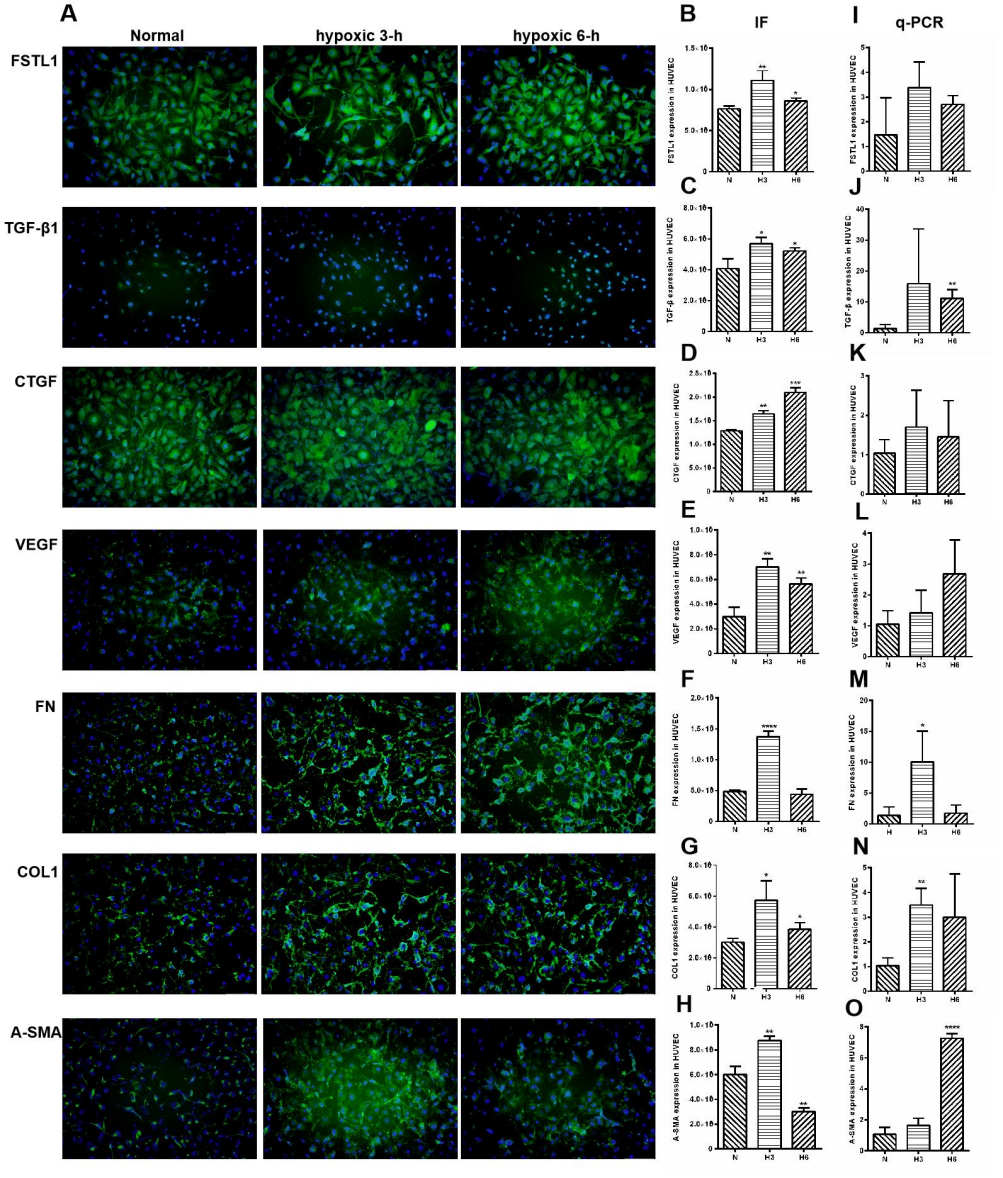
**The expression of FSTL1, TGFβ, CTGF, VEGF, FN, COL1 and α-SMA in HUVECs.** In Immunofluorescence figures, (**A**) indicates normal cell fluorescence, hypoxic 3 h cell fluorescence and hypoxic 6 h cell fluorescence (10×). (**B**–**H**) indicates the quantitative analysis of immunofluorescence figures in the normal, 3 h hypoxia, and 6 h hypoxia models using ImageJ. The 3 h and 6 h hypoxia results indicated that FSTL1, TGFβ1, CTGF, VEGF, FN, COL1, and α-SMA were expressed at significantly higher levels than in normal cells. (**I**–**O**) indicates that the mRNA expression of *FSTL1* and ECM-related factors in the three models. The expression of *FSTL1*, *TGFβ1*, *CTGF*, *VEGF*, *FN*, *COL1*, and *α-SMA* mRNA in 3 h and 6 h hypoxia conditions, respectively, were expressed at significantly higher levels than in normal cells (N: normal, h3: 3 h hypoxia, h6: 6 h hypoxia). Data are expressed as mean ± SEM. * means P < 0.05, **means P < 0.01, *** means P < 0.001.

The expression of FSTL1 and ECM-related factors were analyzed under hypoxic conditions and the results provided a foundation for subsequent FSTL1-related experiments. In the hypoxic model, which included hypoxia for 3 hours and 6 hours, FSTL1, TGFβ1, CTGF, VEGF, FN, COL1, and α-SMA were expressed at significantly higher levels than were observed in normal HUVECs (immunofluorescence, Figure 8A), (*p*<0.05, Figure 8B–8H). These results were consistent with qPCR results (*p*<0.05, Figure 8I–8O).

### The proteins expression of FSTL1 and ECM-related factors in HRCECs and HUVECs under hypoxic conditions

**Figure 9:**
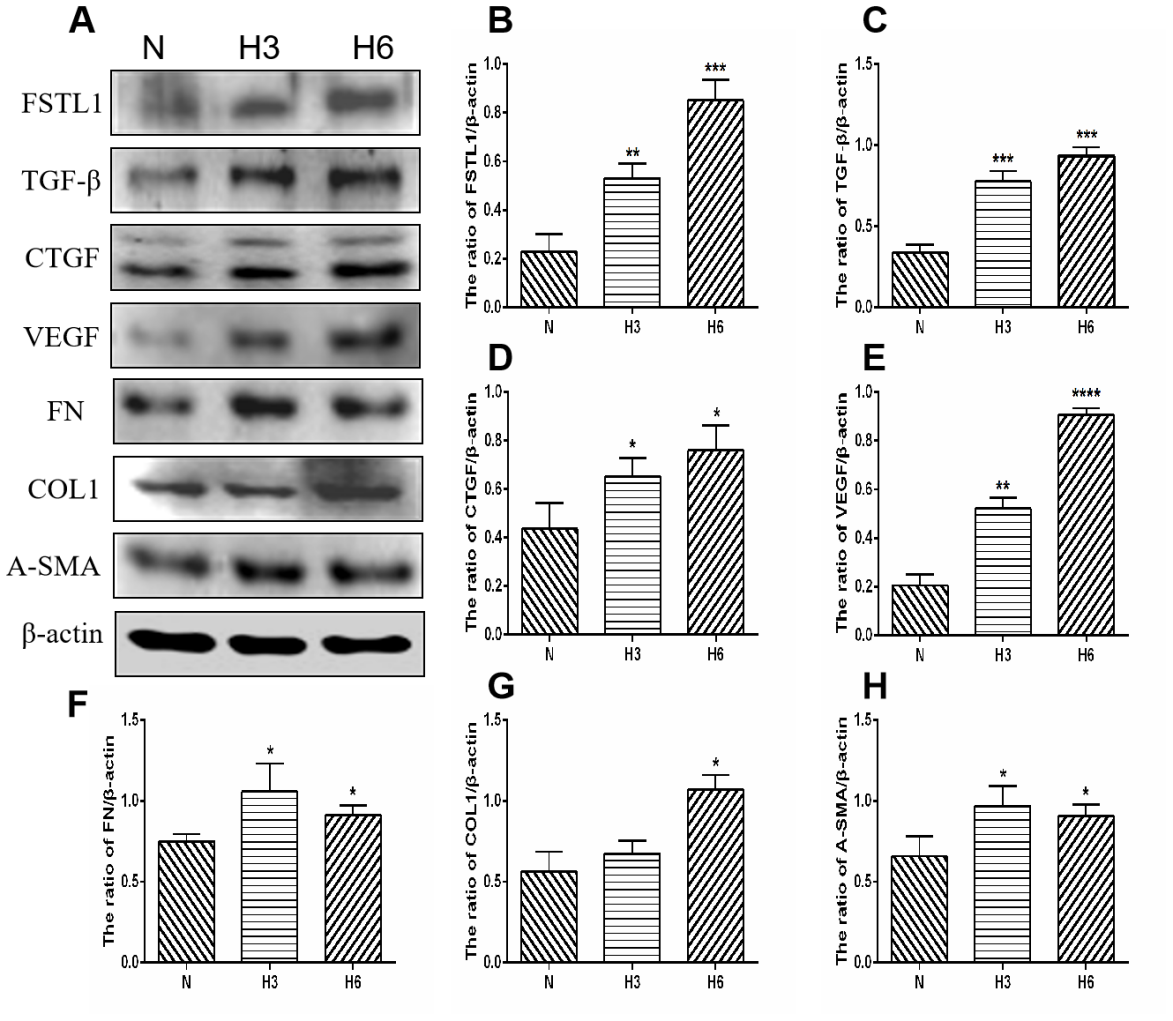
**The expression of FSTL1, TGFβ, CTGF, VEGF, FN, COL1, and α-SMA proteins in HRCECs.** (**A**) shows western blots representatives of FSTL1, TGFβ, CTGF, VEGF, FN, COL1, α-SMA and β-actin in HRCECs. (**B**–**H**) shows the ratio of FSTL1, TGFβ, CTGF, VEGF, FN, COL1, and α-SMA to β-actin in the hypoxic treated HRCECs and normal controls, respectively. Indicates the quantitative analysis of western blots figures in the normal, 3 h hypoxia, and 6 h hypoxia models using ImageJ. The 3 h and 6 h hypoxia results indicated that FSTL1, TGFβ, CTGF, VEGF, FN, COL1, and α-SMA were expressed at significantly higher levels than in normal cells (N: normal, h3: 3 h hypoxia, h6: 6 h hypoxia). Data are expressed as mean ± SEM. * P < 0.05, ** P < 0.01, *** P < 0.001.

**Figure 10:**
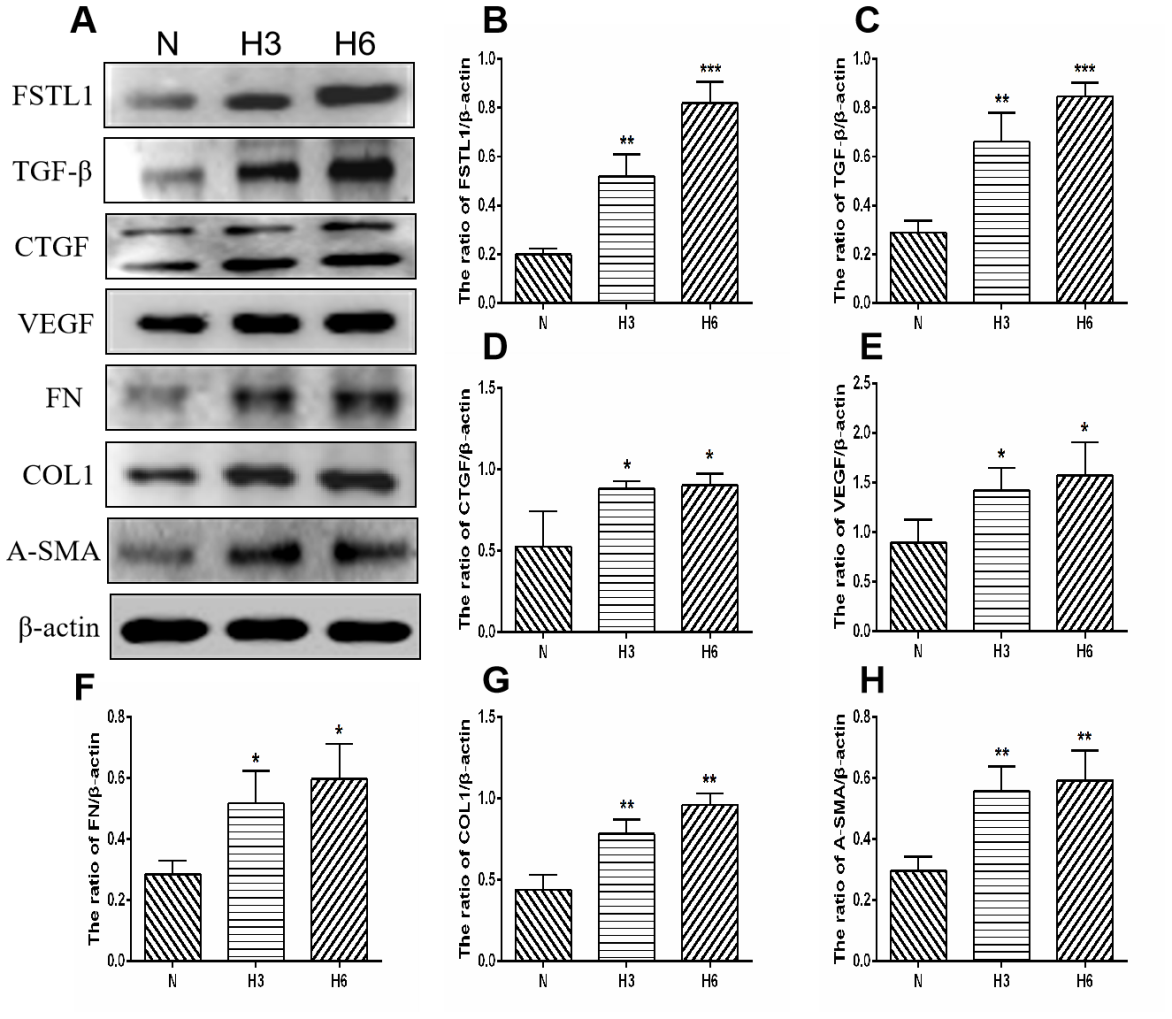
**The expression of FSTL1, TGFβ, CTGF, VEGF, FN, COL1, and α-SMA proteins in HUVECs.** (**A**) shows western blots representatives of FSTL1, TGFβ, CTGF, VEGF, FN, COL1, α-SMA, and β-actin in HUVECs. (**B**–**H**) shows the ratio of FSTL1, TGFβ, CTGF, VEGF, FN, COL1, and α-SMA to β-actin in the hypoxic treated HUVECs and normal controls, respectively. Indicates the quantitative analysis of western blots figures in the normal, 3 h hypoxia, and 6 h hypoxia models using ImageJ. The 3 h and 6 h hypoxia results indicated that FSTL1, TGFβ, CTGF, VEGF, FN, COL1, and α-SMA were expressed at significantly higher levels than in normal cells (N: normal, h3: 3 h hypoxia, h6: 6 h hypoxia). Data are expressed as mean ± SEM. * P < 0.05, ** P < 0.01, *** P < 0.001.

Parallel to the immunofluorescence and qPCR assays, the concentrations of the 7 secreted proteins were significantly increased under hypoxic conditions as compared with those in the normal control group in HRCECs (*p*< 0.05, Figure 9). HUVECs yielded the same results (*p*< 0.05, Figure 10).

### *FSTL1* expression plasmids and the *FSTL1* interference fragment transfected HUVECs

**Figure 11:**
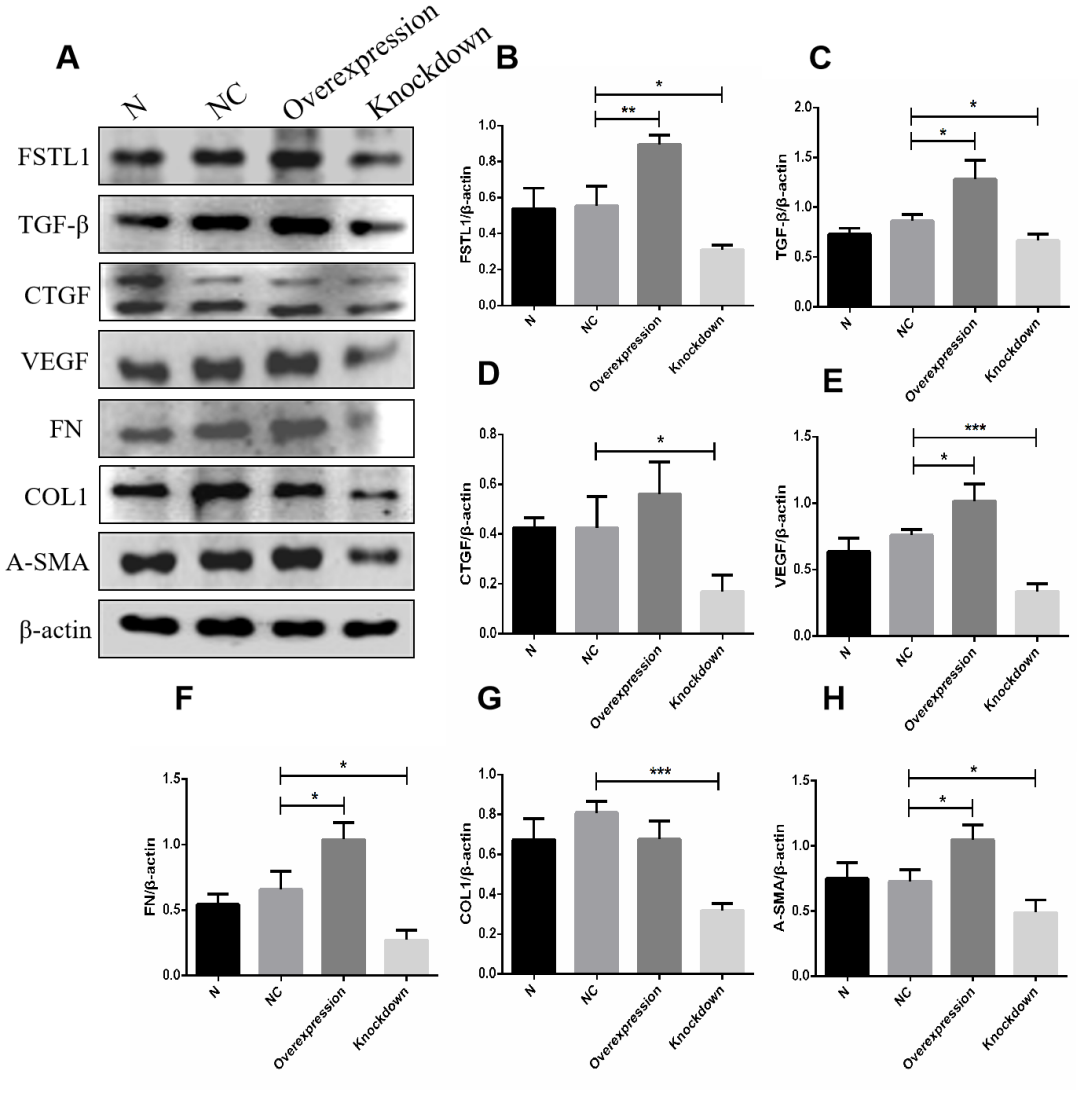
**The expression of FSTL1, TGFβ, CTGF, VEGF, FN, COL1 and α-SMA proteins in *FSTL1* overexpression group and *FSTL1* knockdown group.** (**A**) shows western blots representatives of FSTL1, TGFβ, CTGF, VEGF, FN, COL1, α-SMA and β-actin in HUVECs. (**B**–**H**) shows the ratio of FSTL1, TGFβ, CTGF, VEGF, FN, COL1 and α-SMA to β-actin in the blank control group (N), negative control group (NC), *FSTL1* overexpression group (Overexpression) and *FSTL1* siRNA group (Knockdown) respectively. The western blots figures were quantitative analyzed by ImageJ. FSTL1, TGFβ, VEGF, FN and α-SMA proteins were all upregulated in *FSTL1* overexpression group compared with negative control group, and the expression of FSTL1, TGFβ, CTGF, VEGF, FN, COL1, and α-SMA proteins were all downregulated in *FSTL1* knockdown group compared with negative control group (N: blank control group, NC: negative control group, Overexpression: *FSTL1* overexpression group, Knockdown: *FSTL1* siRNA group). Data are expressed as mean ± SEM. * P < 0.05, ** P < 0.01, *** P < 0.001.

In order to further study the function of FSTL1 on vascular endothelial cells, we constructed *FSTL1* expression plasmids and an *FSTL1* interference fragment to transfect HUVECs. According to the results of western blots and qPCR, the expression of FSTL1 in vascular endothelial cells in the *FSTL1* overexpression group was significantly higher compared with the negative control group (*p*< 0.05, Figure 11A, 11B), suggesting that the *FSTL1* overexpression model was successfully constructed. The expression of FSTL1 in vascular endothelial cells of *FSTL1* knockdown group was significantly lower compared with the negative control group (*p*< 0.05, Figure 11A, 11B), suggesting that the cell model in which *FSTL1* was silenced was successfully constructed.

### The expression of VEGF and other related extracellular matrix (ECM) factors in the *FSTL1* overexpression group and the *FSTL1* knockdown group

In view of the fact that FSTL1 is involved in the pathogenesis of DR. We tried to ascertain the effects of FSTL1 on the vascular endothelial cells by analyzing the expression of VEGF and other related extracellular matrix (ECM) factors using western blots and qPCR in *FSTL1* overexpression group and the *FSTL1* knockdown group. FSTL1, TGFβ, VEGF, FN, and α-SMA protein expression was upregulated in the *FSTL1* overexpression group, FSTL1, TGFβ, CTGF, VEGF, FN, COL1, and α-SMA protein expression was downregulated in the *FSTL1* knockdown group compared with the negative control group (*p*< 0.05, Figure 11).

**Figure 12:**
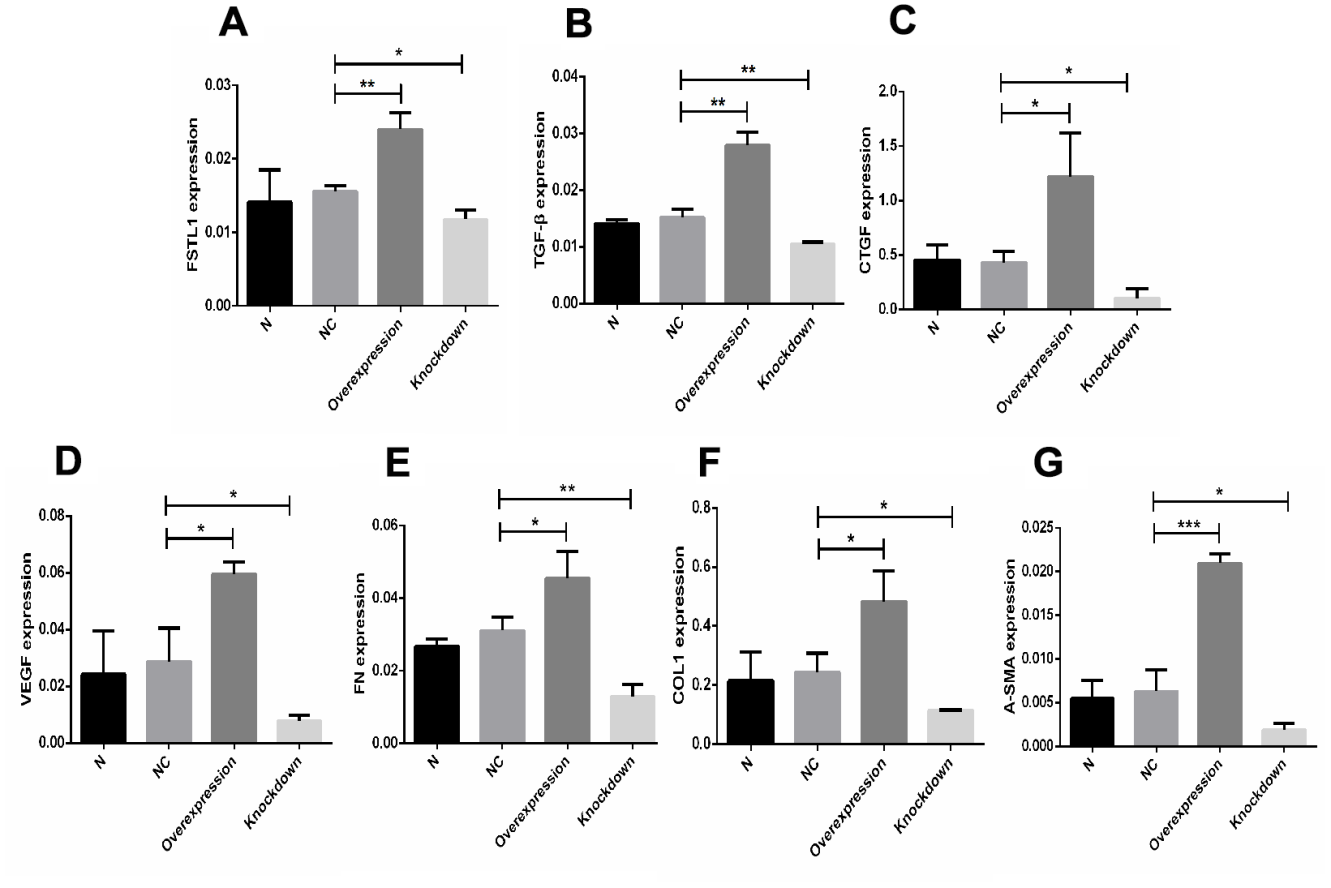
**The expression of *FSTL1*, *TGFβ1*, *CTGF*, *VEGF*, *FN*, *COL1*, and *α-SMA* mRNA in *FSTL1* overexpression group and *FSTL1* knockdown group.** (**A**–**G**) indicates that the *FSTL1* and ECM-related mRNA expression in the blank control group (N), negative control group (NC), *FSTL1* overexpression group (Overexpression) and *FSTL1* siRNA group (Knockdown) respectively. The expression of *FSTL1*, *TGFβ1*, *CTGF*, *VEGF*, *FN*, *COL1*, and *α-SMA* mRNA were all upregulated in *FSTL1* overexpression group compared with negative control group, and were all downregulated in the *FSTL1* knockdown group (N: blank control group, NC: negative control group compared with negative control group, Overexpression: *FSTL1* overexpression group, Knockdown: *FSTL1* siRNA group). Data are expressed as mean ± SEM. * P < 0.05, ** P < 0.01, *** P < 0.001.

*FSTL1*, *TGFβ1*, *CTGF*, *VEGF*, *FN*, *COL1*, and *α-SMA* mRNA expression was upregulated in the *FSTL1* overexpression group, *FSTL1*, *TGFβ*, *CTGF*, *VEGF*, *FN*, *COL1*, and *α-SMA* mRNA expression was downregulated in the *FSTL1* knockdown group compared with the negative control group (*p*< 0.05, Figure 12).

## DISCUSSION

FSTL1 is a TGF-β-induced glycoprotein secreted from parent cells belonging to a family of secreted proteins rich in cysteine with pleiotropic functions [13–16]. The expression of FSTL1 was found limited to non-hematopoietic lineage cells, especially mesenchymal lineage cells [17–21]. FSTL1 is not only involved in a variety of biological activities (including regulating cell survival, proliferation, differentiation, migration, organ development, carcinogenesis, and metastasis, etc.), but also regulates many signaling pathways, and has been shown to bind to DIP2A, TLR4, and BMP receptors [13, 22]. Previous studies suggest that it plays a role in respiratory diseases, cardiovascular system, immune system, and tumor diseases, but research is lacking regarding FSTL1 in ophthalmology [10, 11, 23].

The joint action of CTGF and TGF-β1 induces fibroblasts to become myofibroblasts, resulting in collagen deposition, and ultimately to organ scarring and fibrosis [24]. FSTL1 exerts an important effect on myofibroblasts by positively regulating the TGF-β1 signaling pathway, as well as regulating fibroblast activation, myofibroblast differentiation, and matrix protein production [25]. TGF-β1 upregulates the expression of FSTL1 via the *de novo* synthesis of mRNA, a process that is mainly mediated by TGF-β1/Smad3 signaling [14]. TGF-β1 enhances the synthesis of FN-based ECM molecules through SMAD-mediated CTGF expression [26]. The correlation between the above factors, ECM, and CTGF supports the notion that the detected ECM pathway plays a role in fibrosis, and the relationship between the ECM pathway, FSTL1, and CTGF will be further explored [27–30]. Studies have found that lung injury induces FSTL1 expression, which promotes myofibroblast accumulation and subsequent fibrosis, while blockade of FSTL1 with a neutralizing antibody can inhibit pulmonary fibrosis in mice [15].

FSTL1 may play a pro-angiogenic role in tumor development and angiogenesis (survival and migration) in a manner similar to that shown *in vitro* and in ischemic tissues [31]. According to previous studies, FSTL1 is highly expressed in blood vessels of the developing lung, and plays a key regulatory role in maintaining the normal physiological function of the cardiovascular system [11]. The AKT-VEGF signaling axis is a critical regulator of vascular recruitment in the process of tissue growth. Akt-eNOS signaling is involved in the regulation of endothelial cell function and vascular length under ischemic stress conditions. It has been found that FSTL1 promotes revascularization by activating Akt-eNOS signaling in endothelial cells under chronic ischemic stress [10, 32]. However, the function of FSTL1 in retinal vascular endothelial cells is still unknown.

We found that all sequencing results included *FSTL1* and ECM receptor pathways, and the expression of *FSTL1* was downregulated in the HG + Lu + anti-CTGF group. The expression of FSTL1 appeared greatly increased in IVB-treated membranes and in the vitreous humor of PDR patients with anti-VEGF injection. The above experiments indicated that FSTL1 plays an important role in enhancing fibrosis in PDR. These results provided preclinical data for subsequent experiments and suggested that FSTL1 was related to fibrosis caused by anti-VEGF. We observed that *FSTL1* expression was inhibited after dual-target intervention, and a direct or indirect relationship exists between FSTL1 and CTGF. Therefore, it was necessary to further study its potential regulatory mechanisms. Based on the above experimental results and previous studies, our team focused on FSTL1 as a key point to observe the involvement of regulatory pathways and to explore its relationship with common pro-fibrotic factors.

Present studies show that hyperglycemia can cause retinal cell dysfunction by altering various growth and inflammatory factors, leading to cell damage, retinal ischemia, and hypoxia, which further aggravates DR progress [33, 34]. Therefore, we created a hypoxia model to further investigate the expression of FSTL1 in HRCECs and HUVECS under hypoxic conditions. We found that FSTL1 and fibrotic factors in the hypoxic model were expressed at significantly higher levels than in normal HRCECs and HUVECs. Our results suggested that FSTL1 may play a crucial role in the pathogenesis of PDR.

Interestingly, our research revealed increased fibrosis and FSTL1 appeared up-regulated after treatment with anti-VEGF. Conversely, FSTL1 expression was down-regulated upon treatment with anti-VEGF in combination with anti-CTGF. Thus, we considered that FSTL1 may greatly influence the pathogenesis of PDR, and associates with anti-VEGF-induced fibrosis. The results suggested that FSTL1 expression may be effectively inhibited by anti-CTGF treatment. FSTL1 promotes both fibrosis and angiogenesis based on previous research regarding other diseases, we believe that FSTL1 may serve as a new therapeutic target to treat neovascularization and fibrosis simultaneously, but our research has not yet addressed this aspect. Further studies are required to explore the impact of FSTL1 on neovascularization and fibrosis to better understand the mechanism by which FSTL1 influences PDR [15, 32].

In order to further study the function of FSTL1 on vascular endothelial cells, we constructed *FSTL1* expression plasmids and an *FSTL1* interference fragment to transfect HUVECs to better understand the mechanism through which FSTL1 influences PDR. Our research revealed that TGF-β, VEGF, FN, and α-SMA were all upregulated in *FSTL1* overexpression cells, and the expression of TGF-β, CTGF, VEGF, FN, COL1, and α-SMA were downregulated in the *FSTL1* knockdown group, indicating that there was a mutual regulation relationship among FSTL1, VEGF, and the ECM-related factors. Therefore, FSTL1 may be a new potential therapeutic target for the treatment of both neovascularization and fibrosis in DR.

However, there are several limitations to our research. First, patients present with varied sensitivities to diverse drugs, and it leads to relatively strong individual differences among disparate membranes and vitreous humor. Second, due to technical constraints, the membrane tissue was sliced into diverse layers and stained with different antibodies. The mean fluorescence intensities among different layers cannot be compared. Third, the present sample size was small and subsequent experiments should expand the sample size for further exploration. Fourth, the potential mechanism of FSTL1 on neovascularization and fibrosis requires further investigation.

## MATERIALS AND METHODS

The Ethics Committee and the Institutional Review Board of the Tianjin Medical University Eye Hospital approved the human patient research program based on the Helsinki Declaration. Written informed consent was obtained from each subject. Ethical batch number: 2013KY (L)-10.

### Proliferative membrane immunofluorescence

Eight PDR proliferating membranes were obtained from six samples each in the intravitreal bevacizumab (IVB) treatment group and the untreated group. The samples were formalin-fixed, paraffin-embedded, cut into sections (5 μm thick), and then dewaxed and rehydrated. After the sections were washed with phosphate-buffered saline (PBS), they were incubated with streptavidin-peroxidase for 10 min, washed, and then blocked in goat serum. Sections were incubated with antibodies against the following antigens at 4° C overnight: transforming growth factor-β1 (TGF-β1, 1:250 dilution, BA0290, Bosterbio), CTGF (1:250 dilution, BA0752, Boster), alpha smooth muscle actin (α-SMA, 1:250 dilution, bs-10196R, Bioss), Collagen Type I Alpha 1 (COL1A1, 1:250 dilution, BA0325, Boster), fibronectin (FN, 1:250 dilution, BA1771, Boster), and vimentin (1:800 dilution, ab128507, Abcam). The sections were then washed with PBS and incubated with biotinylated goat anti-rabbit IgG (1:1000 dilution, ab150077, Abcam) for 1 h at room temperature. The samples were counterstained with DAPI and photographed using a fluorescence microscope. Fluorescence intensity was analyzed using image software and quantitative analysis was performed using ImageJ (Version 1.8.0, http://imagej.nih.gov/ij).

### Cell culture and experimental group

Rhesus choroid retinal endothelial cells (RF-6A), kept by the Tianjin Medical University Eye Institute. Cells were cultured with 10000 U/mL penicillin and 10000 μg/mL streptomycin in RPMI-1640 complete medium containing 10% fetal bovine serum (FBS) in an incubator at 37° C, supplied with 5% CO_2_. The cells were divided into three groups: a blank control group of normal cultured retinal vascular endothelial cells, an infection control group treated with a scramble shRNA, and a virus infection group treated with CTGFshRNA.

### Real-time quantitative PCR was used to detect mRNA expression levels of *VEGF* and related extracellular matrix (ECM) proteins

Total RNA was extracted using Trizol (15596-026, Ambion) according to the manufacturer’s instructions, and the concentration was determined using spectrophotometry. An A260/A280 ratio between 1.8 and 2.0 indicated that the RNA quality was good. According to the measured RNA concentration, the corresponding volume that contained 1 μg of RNA was calculated. This volume was added to a sterile nuclease-free 0.2 ml PCR tube placed on ice in advance, and the components required for the reverse transcription reaction were sequentially added. A total reaction volume of 20 μl was used. The mixture was then incubated at 65° C for 5 min, 42° C for 60 min, and 70° C for 5 min to obtain the cDNA product that was used for subsequent PCR reactions. In the amplification reaction, 2 μl of template cDNA, 2 μl of upstream and downstream primers, and 4 μl of SYBR fluorescent dye (final volume of 8 μl) were sequentially added to 384-well plates (operated in the dark). In all, three replicates were run per group, and the experiments were repeated three times. The reaction was carried out in a Real-time PCR instrument (ABI7900HT, ABI) to obtain a corresponding amplification reaction and output CT values. Data analysis was performed according to the delta-delta CT method.

### Construction of cell models

### *Cell cultures and experimental grouping*

The cells were divided into the following three groups: V + Lu group (V: VEGF, Lu: Lucentis, an anti-VEGF drug, VEGF and anti-VEGF drug group), 50 ng/ml VEGF for 72 h with 2.5 μg/ml anti-VEGF for 72 h; HG + Lu group (HG: high glucose, a high glucose and anti-VEGF group), incubated with 25 mmol/L glucose for 48 h, followed by anti-VEGF for 72 h; HG + Lu + anti-CTGF group (a high glucose, anti-VEGF, and anti-CTGF group), incubated with 25 mmol/L glucose for 48 h, followed by anti-VEGF for 72 h, then MOI20 of CTGFshRNA virus for 72 h.

### RNA-Seq sample collection and preparation

### *RNA quantification and identification*

Total RNA was prepared by organic extraction and adsorption onto a silica gel membrane of a spin column. Quality control sequencing data were obtained, including concentration, purity, integrity, and DNA contamination, to ensure that the quality of the subsequent processing data was acceptable. Concentrations were accurately quantified using a Qubit® 2.0 fluorometer. Purity was determined using a NanoPhotometer® spectrophotometer, which measured absorbance (formerly known as the optical density (OD) value) and the A260/280 and A260/230 ratios. Integrity was accurately determined using an Agilent 2100 Bioanalyzer. Potential DNA contamination was determined using agarose gel electrophoresis analysis.

### Library preparation for transcriptome sequencing

Poly(A) RNA enrichment was selected to process total RNA samples. The mRNA was classified according to a degenerate reverse transcription primer with a poly tail A added to the terminal A (i.e., single base anchoring). The resulting poly A tail mRNA was enriched using Oligo (dT) magnetic beads, and the enriched mRNA was randomly interrupted by divalent cations in NEB Fragmentation Buffer. Then, the library was built according to the NEB common database construction method and the chain-specific database construction method. The NEB library was based on fragmented mRNA and random oligonucleotides (used as primers) to synthesize cDNA using the Moloney Murine Leukemia Virus (M-MLV) reverse transcriptase system. To obtain the first strand, RNA strands were degraded using RNase H, and cDNA was synthesized from high-quality deoxyribonucleotides (dNTPs) using a DNA polymerase I system. The purified double-stranded cDNA was then subjected to end repair, a poly-A tail was added, and the sequencing linker was ligated. Approximately 200 base pairs (bp) of cDNA were screened using AMPure XP beads. Thereafter, polymerase chain reaction (PCR) amplification was performed, and the PCR product was purified again using AMPure XP beads to obtain a library.

### Clustering and sequencing

After the library was constructed, a Qubit® 2.0 fluorometer was used for preliminary quantification, and the library was diluted to 1.5 ng/μl. The insert size of the library was then determined using an Agilent 2100 Bioanalyzer. After the insert size was reached, the quantitative concentration of the library was accurately quantified using fluorescence quantitative reverse transcription (qRT)-PCR (the effective concentration of the library was higher than 2 nmol/L) to ensure the quality of the library. Clustering of index-encoded samples was performed on a cBot cluster generation system using a TruSeq PE Cluster Kit v4-cBot-HS (Illumina) according to the manufacturer's instructions. After cluster generation, library preparations were sequenced on an Illumina HiSeq 4000 platform, and paired end reads were generated.

### Data analysis

Low-quality sequences, over-represented sequences, and adaptor sequences were filtered using Bioconductor software (Version 3.60, http//www.bioconductor.org/). Differential expression analysis was performed using the edgeR function, and the protein sequences corresponding to the differentially significant genes (DEGs) with *p* ≤ 0.001 were extracted for Gene Ontology (GO) functional enrichment analysis and pathway analysis. This function was based on the assumption that the expression of each gene obeys a negative binomial distribution, that is, the conditional maximum likelihood method was used to estimate the dispersion of individual genes, and then the empirical Bayesian method was applied to approximate the dispersion to a common value. Finally, two observations were made, and whether the difference between the group mean and 0 was statistically significant was determined. Blast software was used to compare the obtained gene sequences to the NR library and the KEGG (Kyoto Encyclopedia) database, and the GO and pathway annotation information was extracted for all identified genes. The GO and pathway enrichment analyses were performed on DEGs based on the DEG list, the top GO function, and the hypergeometric test in the Bioconductor application. To screen the obtained differential gene data, we filtered out the meaningless data in the range of Log-2 to Log2, found the corresponding genes from the database one-by-one according to the P value, and consulted the relevant literature to understand the role and mechanism of the gene in different tissues.

### Measurement of follistatin-like protein 1 (FSTL1) using ELISA

FSTL1 was measured in vitreous humor obtained from anti-VEGF and non-VEGF-treated PDR patients using a human FSTL1 ELISA kit, according to the manufacturer's instructions. A standard curve was generated using each of the assayed positive protein standards. Each experiment was performed in two wells.

### Proliferative membrane immunofluorescence

Immunofluorescence was performed as previously described. FSTL1 (1:250 dilution, bs-6050R, Bioss), VEGF (1:250 dilution, bs-4572R, Bioss), and vimentin (1:800 dilution, ab128507, Abcam) were used. Fluorescence intensity was analyzed using image software. Quantitative analysis was performed using ImageJ.

### Human retinal capillary epithelial cell (HRCEC) and human umbilical vein endothelial cell (HUVEC) cultures

Human retinal capillary epithelial cell (HRCEC) and human umbilical vein endothelial cell (HUVEC) are both kept by the Tianjin Medical University Eye Institute. Cells were cultured in DMEM (high glucose) with 1000 U/ml penicillin, 10000 μg/ml streptomycin, and 10% FBS in a 37° C incubator supplied with 5% CO_2_. Logarithmic growth phase cells were used for the experiments. In 24-well plates, retinal capillary epithelial cell (HRCECs) and human umbilical vein endothelial cells (HUVECs) were plated at density of 1×10^5^ or 3.8×10^5^ cells per well, respectively, in 6 wells per plate, and were cultured under the following conditions: a hypoxic proliferation model (3 h under nitrogen 24 h after incubation), a hypoxic apoptosis model (6 h under nitrogen 24 h after incubation), and a normal model (95% N_2_, 5% CO_2_).

### Cellular immunofluorescence

HRCECs and HUVECs were fixed in 4% paraformaldehyde in PBS for 10 min at room temperature and then permeabilized with PBS containing 0.1% Triton X-100 for another 10 min. Cells were blocked with bovine serum albumin (BSA) in PBS for 30 min, then incubated with FSTL1 (1:250 dilution, bs-6050R, Bioss), TGFβ1 (1:250 dilution, BA0290, Boster), CTGF (1:250 dilution, BA0752, Boster), VEGF (1:250 dilution, bs-4572R, Bioss), α-SMA (1:250 dilution, bs-10196R, Bioss), COL1A1 (1:250 dilution, BA0325, Boster), and FN (1:250 dilution, BA1771, Boster) antibodies at 4° C overnight. Cells were then washed with PBST and incubated with anti-rabbit secondary antibodies for 1 h at room temperature. The cells were then stained with DAPI for nuclear visualization, and fluorescence intensity was assessed under a microscope by digital analysis. Quantitative analysis was performed using ImageJ.

### Real-time quantitative PCR was used to determine mRNA expression levels of *FSTL1*, *VEGF*, and related extracellular matrix (ECM) proteins

RT-PCR was performed as previously described.

### Western blots

The RAPI protein lysate (R0010, Sangonbio. Co. Ltd) was added to the six-well plate to extract total protein. The BCA method (PC0020, Sangonbio. Co. Ltd) is used for protein quantification. According to the SDS-PAGE loading requirements, add an appropriate amount of sample to the 4-fold loading buffer, boil and denature it at 100° C for 10 minutes. SDS-PAGE electrophoresis, wet transfer method transfer membrane. The membrane was immersed in Western blotting blocking solution II (PC0020, Solarbio. Co. Ltd) (5% skimmed milk powder dissolved in TBST, pH 7.5) shake gently for 1 hour at room temperature. Dilute the primary antibody with TBST β-actin mouse anti-monoclonal antibody (1:1000 dilution, TA-09, ZSGB-BIO. Co. Ltd), FSTL1 (1:1000 dilution, ab223287, abcam), TGF-β (1:1000 dilution, 3711, Cell Signaling), CTGF (1:1000 dilution, 86641,Cell Signaling), VEGFA (1:1000 dilution, ab214424, abcam), FN1 (1:1000 dilution, 26836, Cell Signaling), COL1A1 (1:1000 dilution, 39952, Cell Signaling), α-Smooth (1:1000 dilution, 19245, Cell Signaling) antibody, incubate overnight at 4° C. The membrane was washed 3 times with TBST for 10 minutes each time. Dilute the secondary antibody with TBST, use goat anti-mouse (1:20,000 dilution, ZB2305, HRPZSGB-BIO), goat anti-rabbit HRP (1:20,000 dilution, ZB2301, ZSGB-BIO) (selected according to the primary antibody) and incubate at room temperature for 60 minutes. The membrane was washed 3 times with TBST for 10 minutes each time. After incubation, an exposure scan is performed. The optical density of FSTL1, TGF-β, CTGF, VEGFA, FN1, COL1A1, α-Smooth and β-actin was quantified by Image J (National Institutes of Health, Bethesda, Maryland). Calculate the optical density ratio of FSTL1, TGF-β, CTGF, VEGFA, FN1, COL1A1 and α-Smooth.

### Overexpression plasmid transfection and cell intervention

*FSTL1* expression plasmids (OriGene) and an *FSTL1* interference fragment (Genepharma) were constructed to transfect HUVECs with Lipofectamine 2000 Reagent (52758, Invitrogen) according to the instructions of the manufacturer. The effect of FSTL1 on vascular endothelial cells was clarified by overexpressing *FSTL1* or silencing *FSTL1* expression. The experimental groups were blank control group (N), negative control group (NC), the *FSTL1* overexpression group (Overexpression), and the *FSTL1* siRNA group (Knockdown).

The cell density of logarithmic growth reached about 80%. The Lipor2000 and pcDNA-FSTL1 overexpression vectors or pcDNA-NC vectors were diluted with Opti-MEM (31985-062, Gibco) medium and incubated for 5 min. The diluted Lipor2000 and pcDNA-FSTL1 overexpression vectors or pcDNA-NC vectors were mixed and incubated for 15 min, then added to the cell culture medium and shaken well. After 4–6 h of transfection, the Opti-MEM medium was replaced, and cultures were grown in the complete medium for 24 h. Then, we collected the cells for follow-up experiments. We used the same method to transfect cells with *FSTL1* siRNA. The expression of *FSTL1* in each group was detected by Western blots and real-time quantitative PCR to determine whether the overexpression and silenced *FSTL1* cell models were successfully constructed.

### Western blots and real-time quantitative PCR were used to determine protein and mRNA expression levels of FSTL1, VEGF, and related extracellular matrix (ECM) factors in the *FSTL1* overexpression group and *FSTL1* knockdown group

Western blots and RT-PCR were performed as previously described.

## Supplementary Material

Supplementary Figure 1

Supplementary Table 1
