# Supplementary material for: Follistatin-like protein 1 functions as a potential target of gene therapy in proliferative diabetic retinopathy
Source: Aging (Albany NY). 2021 Mar 10;13(6):8643–64. doi: 10.18632/aging.202678 (PMC8034962; doi:10.18632/aging.202678)
Supplement: Supplementary Figure 1 [file aging-13-202678-s001.pdf]

# SUPPLEMENTARY FIGURE

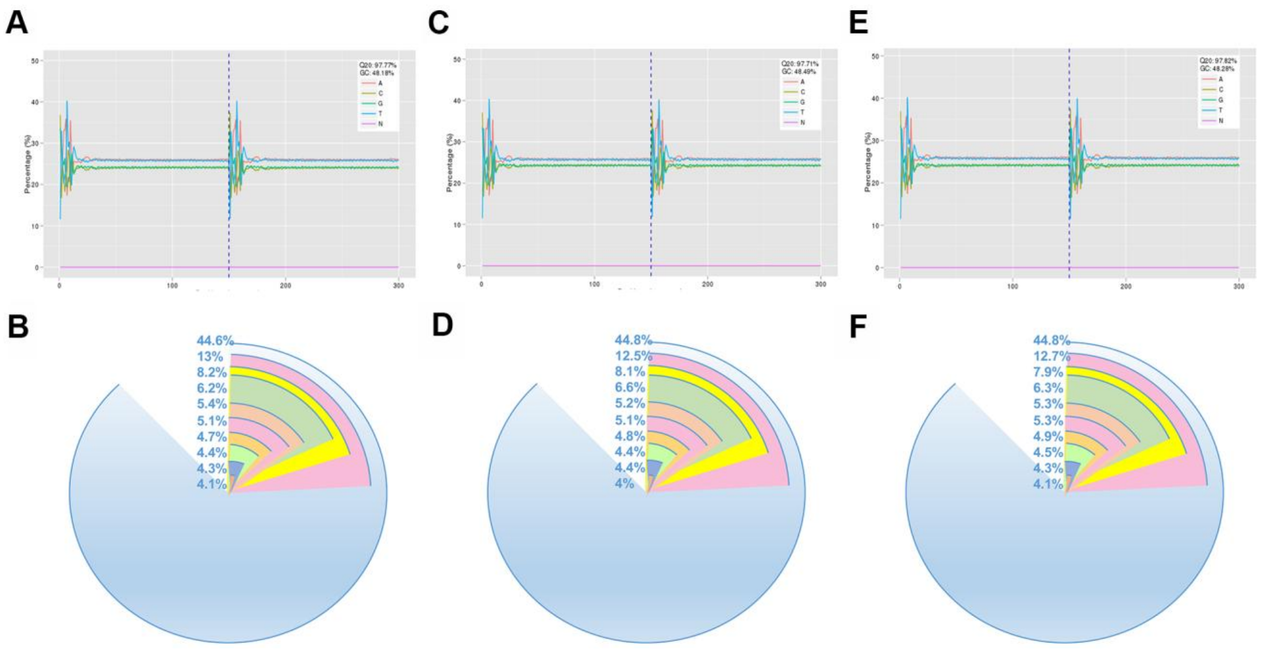


**Supplementary Figure 1. Raw data information and gene coverage.** Transcriptome sequencing of all samples was performed on an Illumina sequencing platform. (**A**, **B**) is the raw data information and gene coverage of HG+LU group. (**C**, **D**) is the raw data information and gene coverage of V+LU group. (**E**, **F**) is the raw data information and gene coverage of HG+LU+anti-CTGF group.
