# Supplementary material for: Follistatin-like protein 1 functions as a potential target of gene therapy in proliferative diabetic retinopathy
Source: Aging (Albany NY). 2021 Mar 10;13(6):8643–64. doi: 10.18632/aging.202678 (PMC8034962; doi:10.18632/aging.202678)
Supplement: Supplementary Table 1 [file aging-13-202678-s002.pdf]

SUPPLEMENTARY TABLE

Supplementary Table 1. Sequencing quality assessment.

Groups	Original reads	Clean reads	Total basepairs	Q20(%)	GC(%)
HG+LU group	49120614	46057708	6908656200	97.78	48.17
V+LU group	49120350	46047754	6907163100	97.83	48.28
HG+LU+anti-CTGF group	49120360	46211118	6931667700	97.72	48.49

Mass analysis indicated that the total number of reads in the HG+Lu group, VEGF+Lu group, and HG+Lu+anti-CTGF group were 49120614, 49120350, and 49120360, respectively. Clean read proportions were 93.76%, 93.74%, and 94.08%, respectively. Among these, the proportions of Q20 (%) and GC (%) were 97.78 and 48.17 in the HG+Lu group, respectively, 97.72 and 48.49 in the VEGF+Lu group, respectively, and 97.83 and 48.28 in the HG+Lu+anti-CTGF group, respectively.
